# Supplementary material for: Association between Immunogenicity of a Monovalent Parenteral P2-VP8 Subunit Rotavirus Vaccine and Fecal Shedding of Rotavirus following Rotarix Challenge during a Randomized, Double-Blind, Placebo-Controlled Trial
Source: Viruses. 2023 Aug 25;15(9):1809. doi: 10.3390/v15091809 (PMC10536230; doi:10.3390/v15091809)
Supplement: Supplementary file 1 [file viruses-15-01809-s001.zip › viruses-2533073-supplementary.pdf]

Supplementary material

Table S1. Baseline demographic and clinical factors between rotavirus shedders and non-shedders, as determined by ELISA, stratified by vaccination status: three injections of P2-VP8-P[8] vaccine (30μg and 60μg doses; n=91) or placebo (n=44)

	**30μg and 60μg P2-VP8-P[8] recipients**			**Placebo recipients**		
	**Not shedding** [a] **(N=76)**	**Shedding** [b] **(N=15)**	**P-value**	**Not shedding** [a] **(N=27)**	**Shedding** [b] **(N=17)**	**P-value**
**Clinical and demographic factors**						
Male sex, n (%)	36 (47.4)	8 (53.3)	0.673	12 (44.4)	7 (41.2)	0.831
Age in days, median (range)	45 (42-55)	45 (42-54)	0.360	44 (42-53)	44 (42-49)	0.751
HIV-exposed, n (%)	10 (13.2)	4 (26.7)	0.237 [c]	4 (14.8)	2 (11.8)	1.000 [c]

ELISA: Enzyme-linked immunoassay; HIV: human immunodeficiency virus
[a] ELISA negative, [b] ELISA positive [c] Fisher's exact test

VISIT WINDOW	PROCEDURES	AGE (RANGE)
Day -7 to -1	Screening<br>Immunogenicity blood draw<br>Safety blood draw	5 – 7 weeks
Day 0	Injection 1 P2-VP8/placebo*	6 – 8 weeks
Day 3 and 7 (±1)	Safety visits<br>Safety blood draw (Day 7)	
Day 28 (-2, +7)	Injection 2 P2-VP8/placebo#	10 – 13 weeks
Day 31 and 35 (±1)	Safety visits	
Day 56 (-2, +7)	Injection 3 P2-VP8/placebo^	14 – 17 weeks
Day 59 and 63 (±1)	Safety visits	
Day 84 (±4)	Rotarix dose 1<br>Immunogenicity blood draw	18 – 22 weeks+
Day 89, 91, 93	Stool collection for Rotarix shedding	18 – 23 weeks
Day 112 (±14)	Rotarix dose 2	22 – 27 weeks
Day 140 (±14)	Rotarix dose 3	26 – 32 weeks
Day 224 (±14)	Final visit<br>Immunogenicity blood draw	9 months (36 – 40 weeks)

Figure S1: Schedule of events for infants.

Note: From Groome, M. J.; Koen, A.; Fix, A.; Page, N.; Jose, L.; Madhi, S. A.; McNeal, M.; Dally, L.; Cho, I.; Power, M.; Flores, J.; Cryz, S., Safety and immunogenicity of a parenteral P2-VP8-P[8] subunit rotavirus vaccine in toddlers and infants in South Africa: a randomised, double-blind, placebo-controlled trial. Lancet Infect Dis 2017, 17, (8), 843-853 (Supplementary Material) [12]. CC-BY-NC.

* Given concomitantly with OPV, Heberbiovac-HB®, Prevenar13® and Pentaxim®
# Given concomitantly with Heberbiovac-HB® and Pentaxim®
^ Given concomitantly with Heberbiovac-HB®, Prevenar13® and Pentaxim®
+ Median age on day 84 is 19 weeks in all treatment groups
